# ATIP3 deficiency facilitates intracellular accumulation of paclitaxel to reduce cancer cell migration and lymph node metastasis in breast cancer patients

**DOI:** 10.1038/s41598-020-70142-7

**Published:** 2020-08-06

**Authors:** Sylvie Rodrigues-Ferreira, Anne Nehlig, Mariem Kacem, Clara Nahmias

**Affiliations:** 1 Université Paris-Saclay, Institut Gustave Roussy, Inserm U981, Biomarqueurs prédictifs et nouvelles stratégies thérapeutiques en oncologie, Gustave Roussy, 94800 Villejuif, France; 2 LabEx LERMIT, University Paris Saclay, 92296 Châtenay-Malabry, France; 3 Inovarion SAS, 75005 Paris, France

**Keywords:** Cancer, Cell biology, Biomarkers, Molecular medicine

## Abstract

Taxane-based chemotherapy is frequently used in neoadjuvant treatment of breast cancer patients to reduce tumor growth and lymph node metastasis. However, few patients benefit from chemotherapy and predictive biomarkers of chemoresistance are needed. The microtubule-associated protein ATIP3 has recently been identified as a predictive biomarker whose low levels in breast tumors are associated with increased sensitivity to chemotherapy. In this study, we investigated whether ATIP3 deficiency may impact the effects of paclitaxel on cancer cell migration and lymph node metastasis. Expression levels of ATIP3 were analyzed in a cohort of 133 breast cancer patients and classified according to lymph node positivity following neoadjuvant chemotherapy. Results showed that low ATIP3 levels are associated with reduced axillary lymph node metastasis. At the functional level, ATIP3 depletion increases cell migration, front-rear polarity and microtubule dynamics at the plus ends, but paradoxically sensitizes cancer cells to the inhibitory effects of paclitaxel on these processes. ATIP3 silencing concomitantly increases the incorporation of fluorescent derivative of Taxol along the microtubule lattice. Together our results support a model in which alterations of microtubule plus ends dynamics in ATIP3-deficient cells may favor intracellular accumulation of paclitaxel, thereby accounting for increased breast tumor sensitivity to chemotherapy.

## Introduction

Breast cancer is a leading cause of death by malignancy in women worldwide. Cancer cell invasion to axillary lymph nodes is a frequent complication of the disease and is associated with poor patient prognosis. Neoadjuvant chemotherapy including taxanes and anthracyclins, administered prior to surgery, is a frequent regimen for a number of breast tumors^1^. However, the proportion of patients who will benefit from chemotherapy remains low, reaching 15–20% in the whole population. The identification of predictive biomarkers, allowing the selection of patients at high risk to resist to chemotherapy, is urgently needed to orient clinicians' decisions. Achievement of pathological complete response (pCR), a surrogate marker of treatment efficiency, is characterized by complete eradication of all invasive cancer cells from the breast and axillary lymph nodes^1,2^, suggesting that chemotherapy not only affects tumor growth but also impairs cancer cell invasion and lymph node metastasis^3^.

Chemotherapeutic drugs such as paclitaxel and docetaxel (taxanes) are microtubule-targeting agents that bind and stabilize microtubules. Initially referred to as “mitotic poisons”, because of their ability to block the mitotic spindle and impair chromosome segregation, taxanes were later shown to suppress microtubule dynamic instability when used at clinically relevant concentrations of the nanomolar range^4^. At very low concentrations, taxanes are no longer able to regulate cell growth but are still effective in reducing cell migration and motility^5–8^. In the search for predictive biomarkers of breast cancer sensitivity to taxane-based chemotherapy, microtubule-associated proteins appeared as good candidates because of their regulatory effects on microtubule assembly, dynamics and functions^9–12^.

In a recent study comparing transcriptional and clinical data from three independent cohorts of breast cancer patients treated with taxane-based chemotherapy, we identified ATIP3—the major product of microtubule-associated tumor suppressor gene *MTUS1*—as a new predictive biomarker of tumor response to chemotherapy^13^. We showed that low levels of ATIP3 in breast tumors correlate with higher sensitivity to taxanes. ATIP3 deficiency sensitizes breast cancer cells to paclitaxel by increasing centrosome amplification and multipolar spindle formation above a critical threshold, which results in a non-viable aneuploid state leading to apoptotic cell death^13,14^. Whether ATIP3 deficiency also sensitizes breast cancer cells to non-mitotic effects of paclitaxel remains to be determined.

ATIP3 has been identified as a potent microtubule stabilizer^15–17^ whose depletion increases microtubule dynamics and cancer cell migration^17^. Low levels of ATIP3 in breast cancer patients are also correlated with increased metastasis and poor clinical outcome^17^. These observations led us to investigate whether ATIP3 deficiency may impact the inhibitory effects of paclitaxel on cell migration and breast cancer metastasis.

We show here that low ATIP3 levels are associated with reduced axillary lymph node metastasis in breast cancer patients following taxane-based chemotherapy, a result that was unexpected given that low-ATIP3 breast tumors are more prone to metastasize^17^. Our data support a mechanism by which ATIP3 silencing favors paclitaxel accumulation on microtubules in interphase cancer cells by increasing the dynamic behavior of growing microtubule plus ends, thereby sensitizing cells to the anti-migratory effects of the drug.

## Results

### Low ATIP3-expressing breast tumors have reduced lymph node metastasis following taxane-based chemotherapy

We first investigated whether expression levels of ATIP3 may be associated with lymph node positivity in breast cancer patients treated with chemotherapy. To this end, we used a breast cancer cohort of 133 patients treated in neoadjuvant setting with taxane-based chemotherapy^18^. For 128 patients, axillary lymph node positivity was sequentially evaluated at diagnosis and after neoadjuvant chemotherapy. Clinical data were compared with expression levels of ATIP3-encoding *MTUS1* gene in naïve tumors (Supplementary Table S1)^13,15,19^.

**Figure 1:**
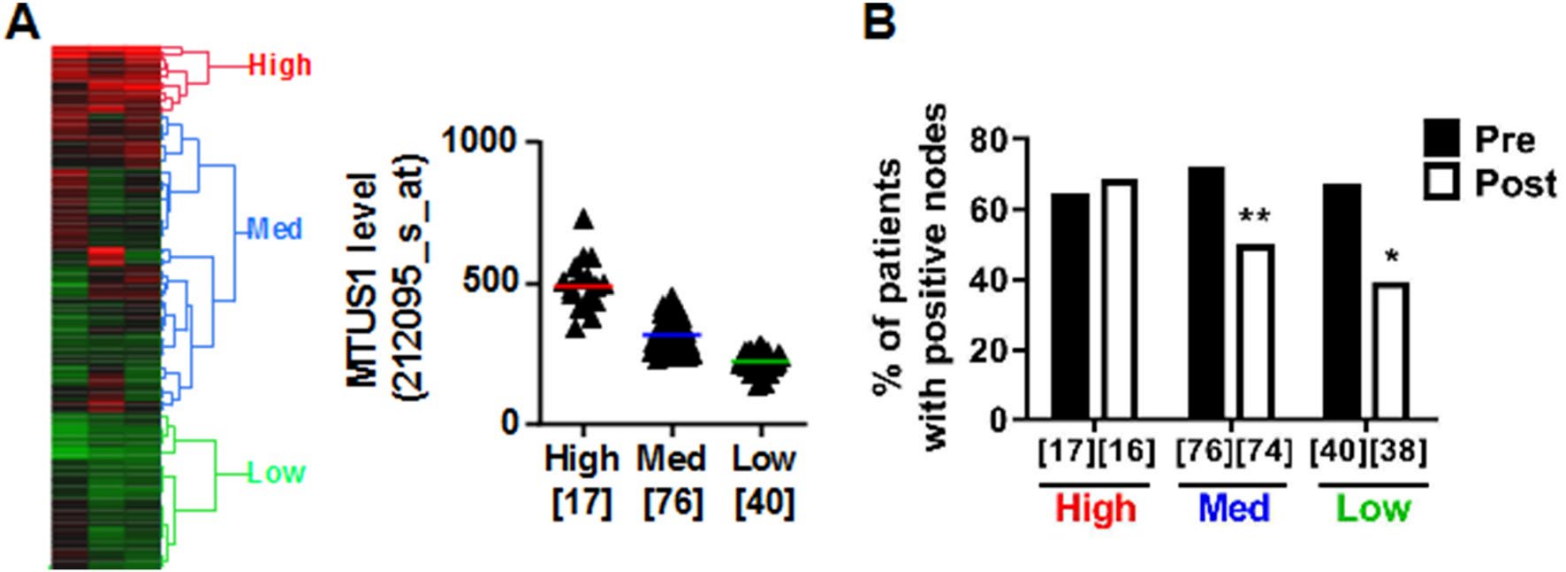
Low ATIP3 tumors have reduced lymph node metastasis after taxane-based chemotherapy. (**A**) Left panel: heat map and hierarchical clustering of 133 breast tumor samples based on the intensities of *MTUS1* (212096_s_at; 212093_s_at; 212095_s_at) probesets. Heat map illustrates relative expression profiles of *MTUS1* (column) for each tumor sample (line) in continuous color scale from low (green) to high (red) expression. Dendogram of the 3 selected tumor groups is shown on the right. Right panel: scattered dot plot of *MTUS1* expression in each of the 3 selected clusters based on the dendogram on the left. Numbers of samples are under brackets. (**B**) Proportion of patients with lymph node metastasis before (pre) and after (post) neoadjuvant taxane-based chemotherapy according to *MTUS1* level in each selected cluster. Number of tumors in each group is indicated under brackets.

Heatmap hierarchical clustering of *MTUS1* Affymetrix probesets intensities extracted from DNA array analysis was used to classify tumors according to low, medium and high *MTUS1* level (Fig. 1A). As shown in Fig. 1B, 65% to 70% of patients had positive lymph nodes before chemotherapy regardless of *MTUS1* level. After taxane-based chemotherapy, the percentage of patients with positive lymph nodes remained unchanged (68.8%) in high *MTUS1*-expressing tumors whereas it decreased to 50% and 39.5% in tumors expressing medium and low levels of *MTUS1*, respectively. Thus, taxane-based chemotherapy is more efficient to prevent axillary lymph node metastasis in patients with low ATIP3-expressing tumors compared to high ATIP3-expressing ones.

### ATIP3 silencing potentiates the effects of paclitaxel on cell migration and polarity

**Figure 2:**
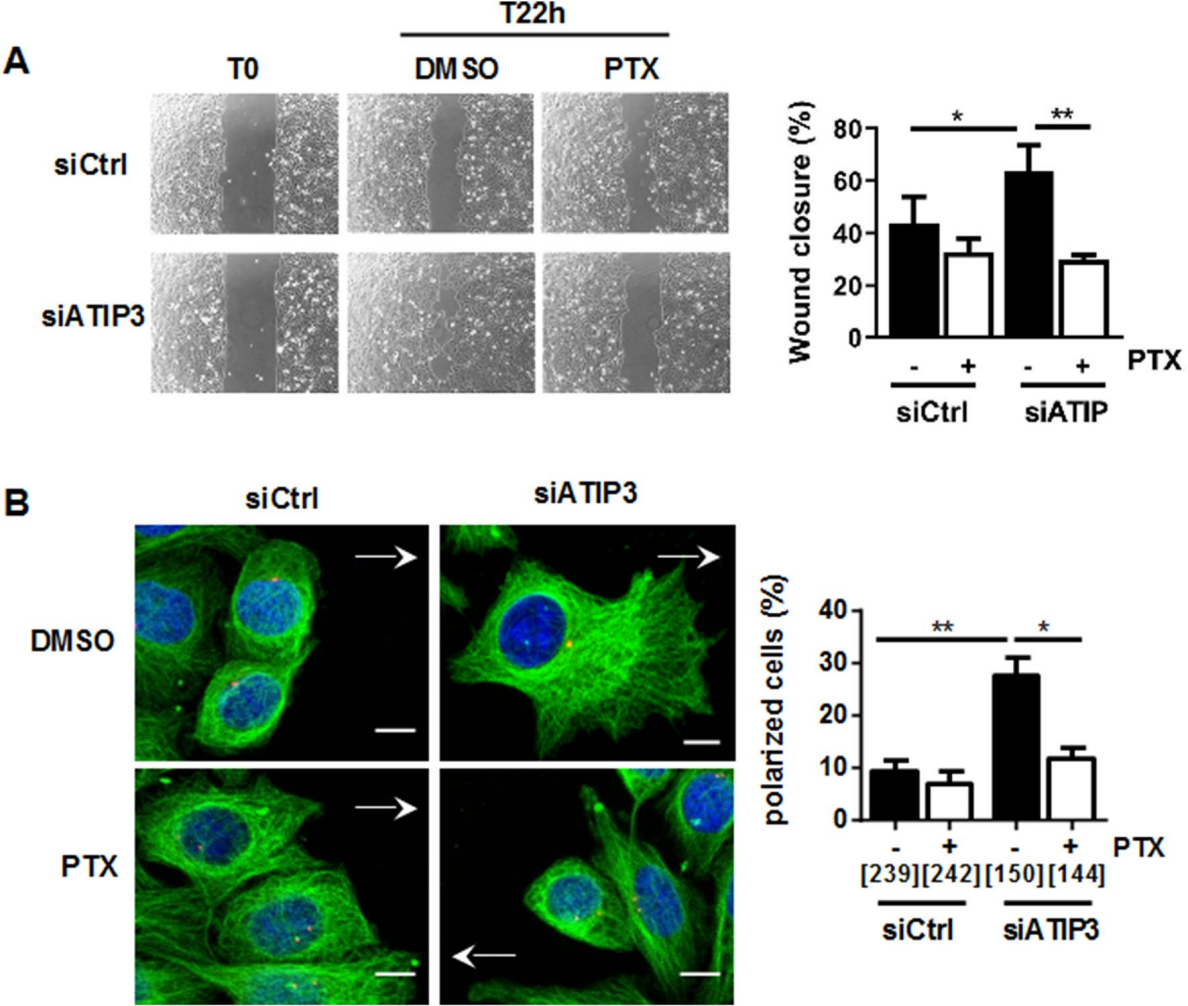
ATIP3 silencing increases PTX effects on cell migration and polarity. (**A**) Migration of HCC1143 breast cancer cells either silenced (siATIP3) or not (siCtrl) for ATIP3 and treated with PTX (1 nM) or vehicle (DMSO). Picture were taken at T0 and after 22 h of migration. Quantification is shown on the right. Shown is one representative experiment out of three performed in quadruplicate. *p < 0.05; **p < 0.01. (**B**) Immunostaining of HeLa cells either silenced (siATIP3) or not (siCtrl) for ATIP3 and allowed to migrate for 3 h in the presence of PTX (1 nM) or vehicle (DMSO). Microtubules were stained in green (anti-alpha-tubulin antibodies), the centrosome in red (anti-pericentrin antibodies) and the nucleus in blue (DAPI). Arrows indicate the direction of migration. Polarized cells are quantified and plotted on the histogram on the right. Numbers of quantified cells are indicated under brackets. Obj × 63, scale bar 10 µM. Shown is one representative experiment out of three performed in triplicate.

Cancer cell spreading to axillary lymph nodes involves cell migration to the metastatic site. We therefore investigated whether ATIP3 deficiency may sensitize cancer cells to the anti-migratory effects of taxanes. Cell migration was studied in two different models of breast cancer cell lines (HCC1143 and MDA-MB-231) exposed to a low dose of paclitaxel (1 nM) that does not affect cell viability (Supplementary Fig. S1A). Cancer cells were ATIP3-depleted using specific siRNA and the consequence of paclitaxel treatment on cell migration was analyzed using a wound healing assay. As shown in Fig. 2A, ATIP3 silencing significantly increased directional migration of HCC1143 cells. Treatment of control cells with a low dose of paclitaxel (1 nM) induced a moderate decrease (11%) in wound closure, that was improved to 34% (*p* = 0.0026) upon ATIP3 silencing. Anti-migratory effects of paclitaxel were also increased from 12 to 65% (*p* = 0.013) in MDA-MB-231 cells upon ATIP3 silencing (Supplementary Fig. S1B), indicating that ATIP3 deficiency improves the effects of paclitaxel on cancer cell migration.

Cells need to polarize in order to migrate in the right direction and be able to close the wound. Polarized cells are characterized by cytoplasmic extension at the leading edge, radial organization of microtubules towards the cell cortex, and positioning of the centrosome between the nucleus and the leading edge^20^. Based on these properties, we analyzed the consequences of ATIP3 silencing on cell polarity in the presence of paclitaxel. As shown in Fig. 2B, ATIP3 silencing markedly increased the percentage of cells that were polarized 3 h after wound scratch. Treatment with paclitaxel at low dose (1 nM) did not significantly modify cell polarity in control cells, but reduced by 51.6% the front-rear polarity in ATIP3-silenced migrating cells (Fig. 2B), further indicating that the inhibitory effects of paclitaxel on cell polarity and migration are improved in ATIP3-silenced cells.

### ATIP3 silencing potentiates the effects of paclitaxel on microtubule dynamics

**Figure 3:**
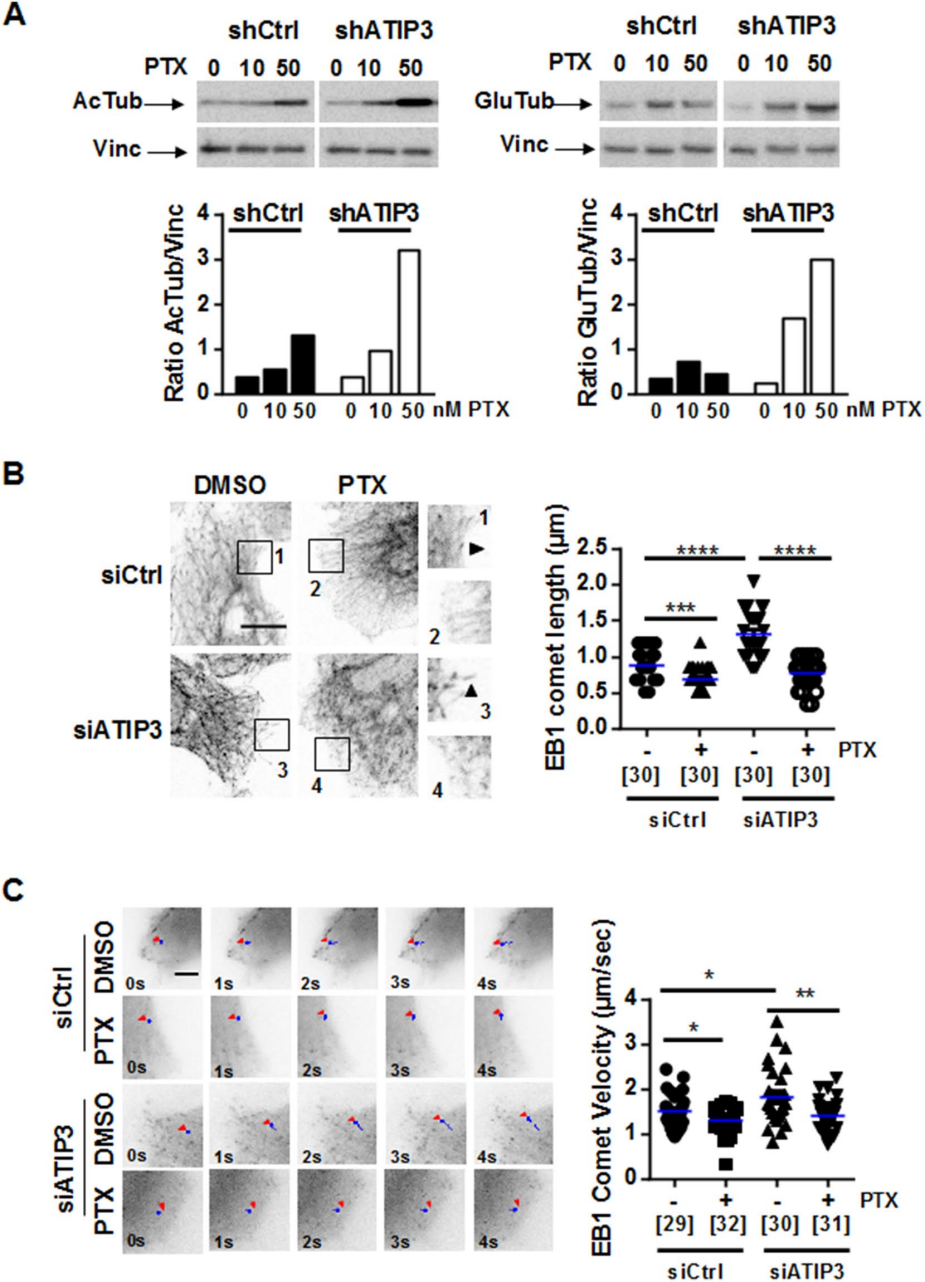
ATIP3 silencing potentiates PTX effects on microtubule stability. (**A**) Western Blot analysis of acetylated tubulin (AcTub, left panel) and detyrosinated tubulin (GluTub, right panel) in SUM52-PE multicellular spheroids expressing (shCrtl) or not (shATIP3) ATIP3, following 3-day treatment with 10 and 50 nM Paclitaxel (PTX). Vinculin (Vinc) is used as internal loading control. Shown is one out of three (left) and four (right) independent experiments. Full-length blots are presented in Supplemental Fig.S2A. Quantification is shown below. (**B**) Representative immunofluorescence photographs of HeLa cells transfected for 48 h with control (siCrtl) or ATIP3-specific (siATIP3) siRNA and treated for 6 h with 5 nM Paclitaxel (PTX). EB1 comets are visualized using rat anti-EB1 antibody. Show is one out of three independent experiments. Comet length was measured and results are plotted in scattered dot plot on the right. Numbers of comets are under brackets. Obj × 63, scale bar 10 µm ***p < 0.001; ****p < 0.0001. (**C**) Representative images of time-lapse videomicroscopy performed on HeLa cells transfected by EB1-GFP and siCtrl or siATIP3 siRNA as indicated, and treated with 5 nM PTX. EB1-GFP comets were tracked to record comet velocity. Results are presented in the scattered dot plot on the right. Numbers of tracked comets are under brackets. Obj × 100, scale bar 5 µm. *p < 0.05; **p < 0.01.

Cell polarity and migration require rapid reorganization of the microtubule network, which relies on its dynamic properties. Paclitaxel is known as a potent microtubule stabilizer. ATIP3 was also reported to stabilize microtubules^17^, leading us to examine microtubule stability upon paclitaxel treatment of ATIP3-silenced cells. Levels of acetylated tubulin (AcTub) and detyrosinated tubulin (GluTub), two markers of stabilized microtubules, were evaluated by Western blotting in ATIP3-positive and -negative SUM52-PE breast cancer cells grown in 3-dimensions. As shown in Fig. 3A, the levels of acetylated tubulin and detyrosinated tubulin were dose-dependently increased in the presence of paclitaxel. They reached a 2.5- and 6.8-fold increase following treatment with 50 nM paclitaxel in ATIP3-positive and ATIP3-negative cells, respectively. Similar results were obtained in 2-dimensional cultures of MDA-MB-231 breast cancer cells (Supplementary Fig. S2), indicating that ATIP3 silencing potentiates the microtubule-stabilizing effects of paclitaxel.

We then evaluated the consequence of ATIP3 silencing on paclitaxel-induced regulation of microtubule dynamics at growing plus ends. For that purpose, we analyzed the length and velocity of End Binding protein (EB1) comets, which are considered as surrogate markers of microtubule growth^6,21,22^. Immunofluorescence studies were conducted in HeLa cells, which are appropriate cellular models suitable for detection of individual microtubule plus ends. As shown in Fig. 3B, treatment with low doses of paclitaxel reduced EB1 comets length by 21% in ATIP3-positive and 42% in ATIP3-negative cells, indicating that paclitaxel is more efficient in conditions of ATIP3 depletion. Time-lapse total internal reflection fluorescence (TIRF) videomicroscopy analyses of EB1-GFP comets were performed in HeLa cells that were ATIP3-depleted or not, and exposed to 5 nM paclitaxel or vehicle, as shown in Supplementary Movies 1 to 4. Subsequent microtubule tip-tracking allowed to measure EB1 comets velocity, which represents microtubule growth rate^6,22^. As shown in Fig. 3C, microtubule growth rate was reduced by 14% in ATIP3-positive cells and 24% in ATIP3-negative cells exposed to paclitaxel, further indicating that ATIP3-deficiency facilitates the effects of paclitaxel on microtubule plus end dynamics.

### ATIP3 silencing facilitates intracellular accumulation of paclitaxel

**Figure 4:**
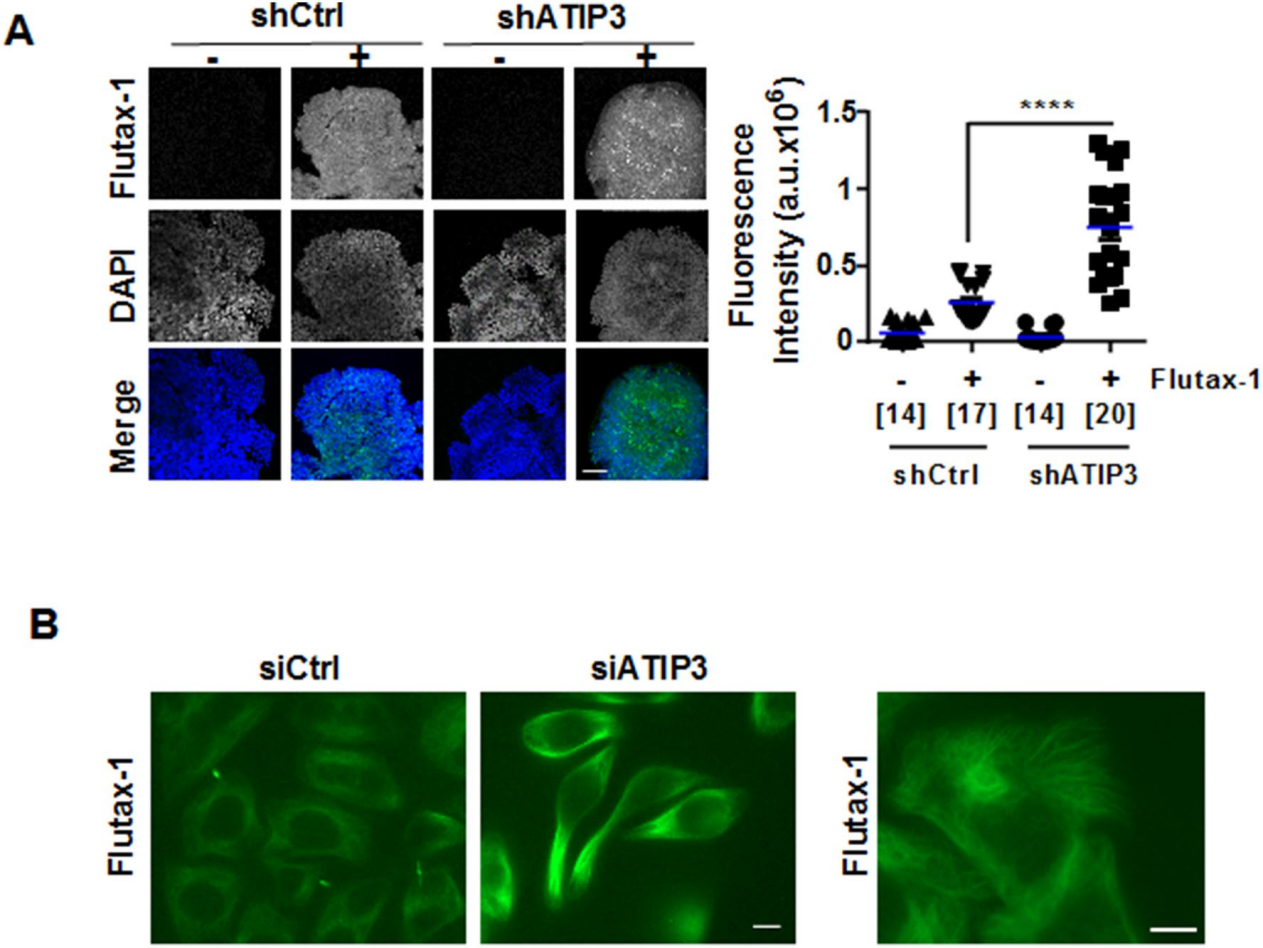
ATIP3 silencing increases intracellular paclitaxel accumulation. (**A**) Representative photographs of fluorescent Taxol derivative Flutax-1 (green) incorporated into SUM52-PE spheroids expressing (shCtrl) or not (shATIP3) ATIP3. DNA was stained in blue (DAPI). Green fluorescence (maximal intensity) was measured in 2 to 5 fields (150 × 150 pixels) for each individual spheroid and results are presented in scattered dot plot on the right. Numbers of fields are under brackets. Obj × 20. a.u. arbitrary units. Scale bar 100 µm. ***p < 0.0001. (**B**) Representative photographs of living HeLa cells expressing (siCtrl) or not (siATIP3) ATIP3 and incubated with fluorescent Taxol derivative Flutax-1 (green). Right panel: Magnification of siCtrl cells shown on the left, revealing that Flutax-1 decorates the microtubule cytoskeleton. Scale bar 10 µm.

To investigate at the molecular level how ATIP3 depletion improves paclitaxel effects on microtubule dynamics, cell polarity and migration, we explored the ability of Flutax-1, a fluorescent derivative of paclitaxel, to accumulate inside the cells following ATIP3-depletion by siRNA. Confocal microscopy was used to measure Flutax-1 incorporation in SUM52-PE breast cancer cells grown in 3-dimensions as multicellular spheroids to mimic tumor architecture. As shown in Fig. 4A, Flutax-1 fluorescence intensity was markedly increased upon ATIP3-depletion. Similar results were obtained using multicellular spheroids from breast cancer cell line HCC1143 (Supplementary Fig. S3). We then turned to 2-dimensional cultures of HeLa cells, that are more suited to examine individual microtubules and Flutax-1 intracellular distribution. As shown in Fig. 4B (left panel), Flutax-1 decorated the microtubule cytoskeleton and intercellular brigdes in living cells. Fluorescence intensity was markedly increased upon ATIP3 depletion. Image magnification (Fig. 4B, right panel) further indicated that Flutax-1 staining decorates the microtubule lattice. Together, these results indicate that intracellular accumulation of paclitaxel is higher in ATIP3-silenced cells compared to control cells, a finding that may account for increased anti-migratory effect of the drug in ATIP3-deficient cells.

## Discussion

The microtubule-associated protein ATIP3 is a predictive biomarker of breast cancer resistance to taxane-based chemotherapy, its deficiency being associated with increased pathological complete response to treatment in breast cancer patients^13^. We show here that low ATIP3 levels in breast tumors are also associated with reduced axillary lymph node metastasis following taxane-based chemotherapy. Accordingly, ATIP3-deficient cancer cells are more sensitive to the inhibitory effects of paclitaxel on cell migration and front-rear polarity. Mechanistically, ATIP3 silencing facilitates the intracellular accumulation of paclitaxel, which may account for increased sensitivity of cancer cells to treatment.

ATIP3 is a potent microtubule stabilizer and its silencing increases cell polarity, migration and microtubule dynamics^17^, all effects that are opposed to those induced by taxanes. Yet, paradoxically, ATIP3 deficiency is shown to improve the effects of paclitaxel rather than resist to the drug. Our results indicate that increased dynamics at microtubule plus ends in ATIP3-depleted cells is associated with increased accumulation of paclitaxel along the microtubule lattice. This is consistent with recent in vitro studies^23^ showing that perturbations of microtubule dynamic parameters, and conformational changes at growing ends, promote taxane binding and microtubule stabilization. Paclitaxel accumulates close to microtubule ends and may then propagate in microtubule lattices^23^. In line with these studies it was recently shown that Carba1, a microtubule-destabilizing agent that modifies plus ends dynamics by increasing catastrophes, synergizes with low doses of paclitaxel by facilitating the incorporation of paclitaxel on the microtubule shaft^24^.

In agreement, End Binding proteins (EB1 and EB3), that bind with high affinity to growing microtubule ends and increase their dynamic behavior^25,26^ have been shown to sensitize cells to taxanes used at nanomolar concentrations^7,8^. Of note, ATIP3 directly interacts with EB1 and prevents its accumulation at microtubule plus ends^27,28^. In ATIP3 depleted cells, increased EB1 binding at growing microtubule ends, and subsequent modifications in dynamic instability parameters, may account for increased incorporation of paclitaxel along the microtubule shaft. Together these data support a mechanism by which ATIP3 deficiency may induce a conformational change of growing microtubule ends to facilitate paclitaxel binding to the lattice, thereby sensitizing cancer cells to the anti-migratory effects of low doses of chemotherapy. This raises the interesting possibility that high levels of EB1 in ATIP3-low breast tumors may be associated with higher sensitivity to anti-migratory effects of paclitaxel, in line with our recent studies showing that combinatorial expression of ATIP3 and EB1 is a prognostic biomarker of breast cancer patient survival^29,30^.

It is to note that ATIP3 deficiency is associated with increased breast cancer metastasis and poor patient survival^17^, and yet favors the reduction of lymph node positivity following taxane-based chemotherapy. This apparent discrepancy is reminiscent of the well-known “triple-negative paradox”^31,32^, in that breast tumors of the triple-negative subtype achieve higher response rates to chemotherapy but remain of worse prognosis.

In conclusion, we show here that ATIP3 deficiency in breast cancer facilitates paclitaxel accumulation on the microtubule lattice of interphase cells, and improves the anti-migratory and anti-metastatic effects of the drug. This, together with our recent report that ATIP3 depletion potentiates the anti-mitotic effects of paclitaxel^13,14^, may account for the potent value of ATIP3 as a predictive biomarker of taxane-based chemotherapy. In the rapidly growing area of personalized medicine, these results open new perspectives for the stratification of breast cancer patients with low ATIP3 levels who may benefit from therapeutic de-escalation. Furthermore, the finding that axillary lymph node metastasis is eradicated by chemotherapy in a substantial percentage of low-ATIP3 breast tumors paves the way to important future clinical studies.

## Methods

### Patients

The M.D. Anderson (MDA) breast carcinoma cohort of patients treated with neoadjuvant chemotherapy was described previously^18^. All patients (n = 133) received weekly paclitaxel and Fluorouracil-Doxorubicin-Cyclophosphamide chemotherapy. Clinical data for lymph node status at diagnosis and after chemotherapy was available for 128 patients. The expression level of *MTUS1* was established using the intensities of three specific *MTUS1* probesets (212093_s_at; 212095_s_at; 212096_s_at) from U133A Affymetrix DNA array study as described^13,18^ and was compared to lymph node status (Supplementary Table S1).

### Cell lines

The human breast cancer cell line SUM52-PE was purchased from Asterand Bioscience (UK) and kindly provided by Dr. Nicholas Turner (Royal Marsden Hospital and Institute of Cancer Research, London, UK). HeLa cells were provided by Dr. Mounira Amor-Gueret (Institut Curie, Orsay, France). HCC1143 breast cancer cells were purchased from American Type Culture Collection (ATCC). MDA-MB-231-Luc-D3H2LN breast cancer cells (designated here MDA-MB-231) obtained from Caliper Life Science (Xenogen) were derived from an in vivo*-*selected metastatic subclone of MDA-MB-231 cells expressing luciferase and grown as described previously^33^.

All cells were used at passages 2 to 20 after thawing and grown as described by the provider. Cells were routinely authenticated by morphologic observation and tested for absence of mycoplasma contamination using MycoAlert Assay detection kit (Lonza).

SUM52-PE cells were silenced for ATIP3 by lentiviral infection of specific ATIP3 shRNA viral particles (Santa Cruz) as described^13^. Silencing was validated by real-time RT-PCR and Western Blot analyses.

### Wound healing

HCC1143 cells were transfected with control-siRNA (20 nM) or ATIP3-specific-siRNA (20 nM) for 48 h and seeded in IBIDI (Biovalley) wound healing chambers. After 24 h the chambers were removed and the wounds were photographed (T0). Cells were allowed to migrate for 22 h to close the wound and then photographed (T22). Wound closure was quantified as previously described^33^.

### Cell polarity assay

HeLa cells were transfected for 24 h with 20 nM control (siCtrl) or ATIP3-specific (siATIP3) siRNA and allowed to migrate for 3 h before they were fixed with ice-cold methanol and incubated with mouse anti-tubulin (Sigma) and rabbit anti-pericentrin (Abcam). Imaging was performed using a Leica SPE confocal microscope with 40X oil objective. Cells were considered polarized when microtubules projected radially, with centrosome located at the front and nucleus at the back of the cell.

### Confocal microscopy

For analysis of EB1 comets, HeLa cells were transfected as above and treated with DMSO or PTX (5 nM) during 6 h prior to immunostaining with mouse anti-tubulin (Sigma) and rat anti-EB1 (Thermofischer) antibodies. Imaging was performed using a Leica SPE confocal microscope with 63X oil objective. Linescan analyses of EB1 fluorescence intensity were performed on a line along the length of microtubule plus end. For quantification of comet number, 10 comets of at least 5 single cells were analyzed in 3 different images.

### TIRF videomicroscopy

HeLa cells were transfected for 24 h with 20 nM control siRNA (siCtrl) or ATIP3-specific-siRNA (siATIP3) using Lipofectamine 2000 (Invitrogen), then transfected for 24 h with 4 µg EB1-GFP construct as described^17^. Transfected cells were treated with DMSO or paclitaxel (5 nM) during 2h30 before being imaged on a Nikon Eclipse Ti microscope with the PFS (Perfect Focus System), equipped with a Nikon CFI Apo TIRF 100X 1.49 N.A. oil objective and controlled with MetaMorph software. Images were acquired in a stream mode at 25 ms exposure time during 30 s. Image analysis was performed with ImageJ software, version 1.51 k (W. Rasband, NIH, USA) and statistical analyses were done with Prism 6.0 (GraphPad software, USA).

### Multicellular spheroids (MCS)

SUM52-PE breast cancer cells (2,000) were seeded in 96-well plate round bottom coated with Polyhema (Poly2-hydroxyethylmethacrylate, Santa Cruz) to prevent cell adhesion. After centrifugation for 5 min at 1,200 rpm, cells were allowed to grow in complete medium. Multicellular tumor spheroids (MCS) were formed after 3 days.

### Flutax-1 incorporation

Breast cancer SUM52-PE grown in 3-dimensions as MCS were incubated with 1 µM Flutax-1 for 48–72 h, then fixed with 4% PAF for 30 min at room temperature. Flutax-1 incorporation in MCS was visualized by confocal microscopy and green fluorescence (maximal intensity) was quantified with ImageJ software. HeLa cells grown in 2-dimensions were incubated with 500 nM Flutax-1 for 18 h and fluorescence was imaged in living cells.

### Antibodies and reagents

Antibodies directed against acetylated-tubulin (611B1) and Vinculin (hVIN-1) were from Sigma, anti-detyrosinated Tubulin (Glu-Tub) was from Millipore, anti-pericentrin was from Abcam and anti-EB1 (KT51) was from ThermoFischer. Paclitaxel (PTX) was purchased from Sigma-Aldrich. Flutax-1 was purchased from R&D Systems.

### Statistical analysis

Significance of the differences between groups were analyzed by One-way ANOVA and Tukey's multiple comparison test using GraphPad 6.0 software. Differences between groups of breast cancer patients were analysed by Chi2 and Fischer's exact test. *p* < 0.05 was considered statistically significant.

## Supplementary information

Supplementary Information 1.

Supplementary Information 2.

Supplementary Information 3.

Supplementary Information 4.

Supplementary Information 5.
