# Supplementary material for: ATIP3 deficiency facilitates intracellular accumulation of paclitaxel to reduce cancer cell migration and lymph node metastasis in breast cancer patients
Source: Sci Rep. 2020 Aug 6;10:13217. doi: 10.1038/s41598-020-70142-7 (PMC7411068; doi:10.1038/s41598-020-70142-7)
Supplement: Supplementary file 5 — Supplementary Information 5. [file 41598_2020_70142_MOESM5_ESM.pdf]

Supplementary Information

ATIP3 deficiency facilitates intracellular accumulation of paclitaxel to reduce cancer cell migration and lymph node metastasis in breast cancer patients

Sylvie Rodrigues-Ferreira, Anne Nehlig, Mariem Kacem, and Clara Nahmias

Legends to Supplementary Figures

Figure S1. ATIP3 silencing potentiates the effects of PTX on MDA-MB-231 cell migration

A- Viability assay performed on HCC1143 (left panel) and MDA-MB-231 (right panel) breast cancer cells silenced (siATIP3) or not (siCtrl) for ATIP3 and treated (+) or not (-) for 24 h with 1 nM paclitaxel (PTX). Data were normalized relative to the value of untreated cells (lane -).

B- Migration of MDA-MB-231 breast cancer cells silenced (siATIP3) or not (siCtrl) for ATIP3 in the presence of PTX (1 nM) or vehicle (DMSO). Pictures are taken at T0 and after 11h of migration (T11h). Quantification is shown on the right. *$p<0.05$.

Figure S2. ATIP3 silencing increases the microtubule-stabilizing effects of PTX

A- Uncropped images from Western Blot analysis of acetylated tubulin (AcTub, left panel) and detyrosinated tubulin (GluTub, right panel) in SUM52-PE multicellular spheroids expressing (shCrtl) or not (shATIP3) ATIP3, following 3-day treatment with 10 and 50 nM Paclitaxel (PTX).

B- Western Blot analysis of acetylated tubulin (AcTub, left panel) and detyrosinated tubulin (GluTub, right panel) in 2-dimensional cultures of MDA-MB-231 breast cancer

cells expressing (siCrtl) or not (siATIP3) ATIP3, following 24 h treatment with 0.2, 1 and 10 nM Paclitaxel (PTX) as indicated. Vinculin (Vinc) is used as internal loading control. Upper panel: uncropped images. Middle panel: cropped images. Lower panel: quantification. Data were normalized relative to the value of untreated cells (lane 0).

Figure S3. ATIP3 silencing improves the intracellular accumulation of fluorescent Taxol derivative Flutax-1

Representative photographs of fluorescent Taxol derivative Flutax-1 (green) staining in 3-dimensional cultures (MCS) of HCC1143 cells expressing (shCtrl) or not (shATIP3) ATIP3. DNA was stained in blue (DAPI). Fluorescence intensity was measured in each individual MCS and results are plotted in scattered dot plot on the right. a.u : arbitrary units. Obj x20. Scale bar 100µM. *** p=0.0003.

Table S1. *MTUS1* level and lymph node status in breast cancer patients

The intensities of three *MTUS1* probesets (212093_s_at; 212095_s_at; 212096_s_at) were used to determine *MTUS1* level. Lymph nodes were evaluated as negative (N0) or positive (N+) before (Pre) and after (Post) neoadjuvant taxane-based chemotherapy. nd : not determined

Supplementary Videos

Movie 1. Time-lapse videomicroscopy of EB1-GFP comets in control HeLa cells treated with DMSO.

Movie 2. Time-lapse videomicroscopy of EB1-GFP comets in control HeLa cells treated with PTX (5 nM).

Movie 3. Time-lapse videomicroscopy of EB1-GFP comets in ATIP3-silenced HeLa cells treated with DMSO.

Movie 4. Time-lapse videomicroscopy of EB1-GFP comets in ATIP3-silenced HeLa cells treated with PTX (5 nM).

Rodrigues-Ferreira et al., supplemental Fig S1

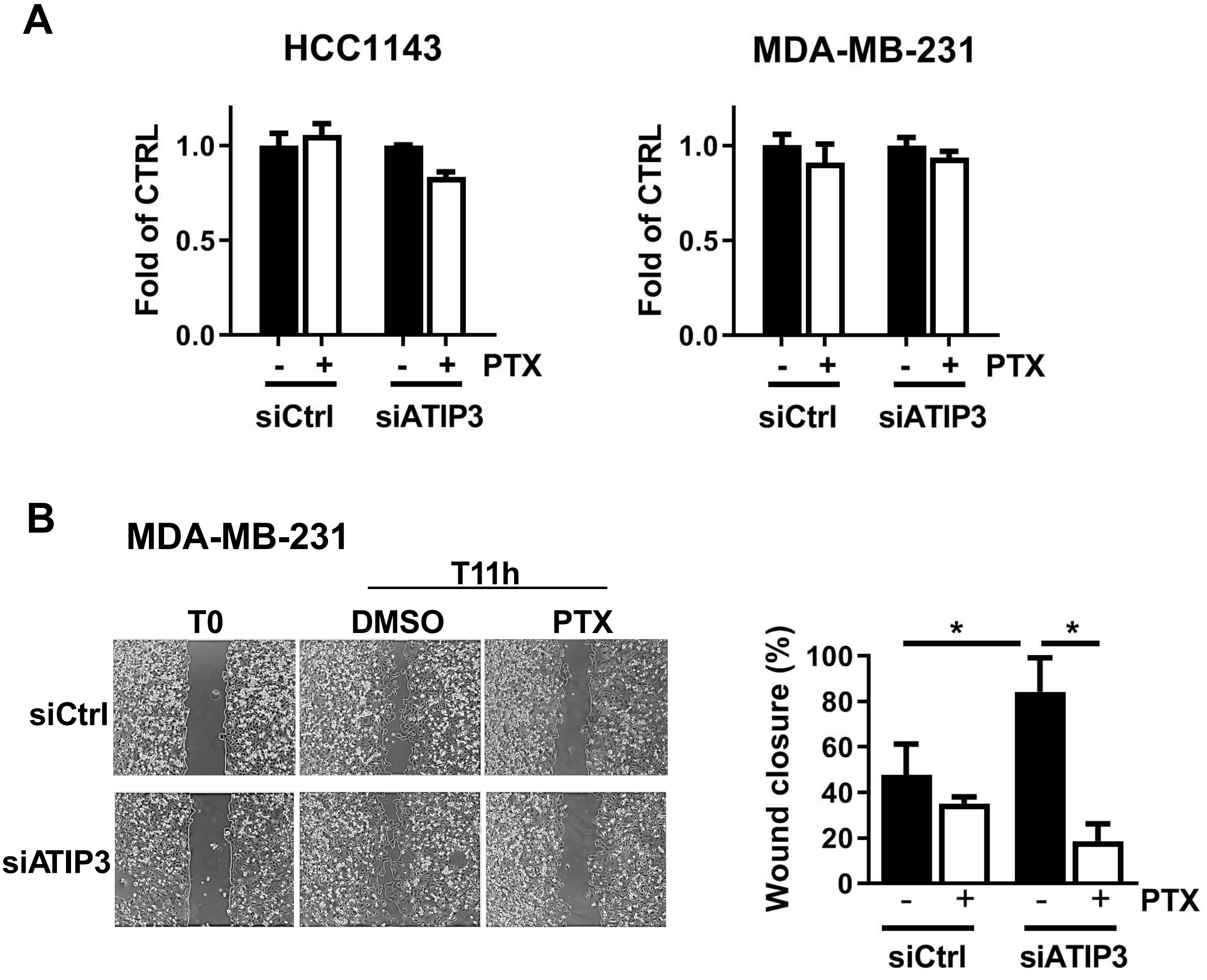

Figure S1. ATIP3 silencing potentiates the effects of PTX on MDA-MB-231 cell migration

A- Viability assay performed on HCC1143 (left panel) and MDA-MB-231 (right panel) breast cancer cells silenced (siATIP3) or not (siCtrl) for ATIP3 and treated (+) or not (-) for 24 h with 1 nM paclitaxel (PTX). Data were normalized relative to the value of untreated cells (lane -).

B- Migration of MDA-MB-231 breast cancer cells silenced (siATIP3) or not (siCtrl) for ATIP3 in the presence of PTX (1 nM) or vehicle (DMSO). Pictures are taken at T0 and after 11h of migration (T11h). Quantification is shown on the right. *p<0.05.

Rodrigues-Ferreira et al., supplemental Fig S2

A

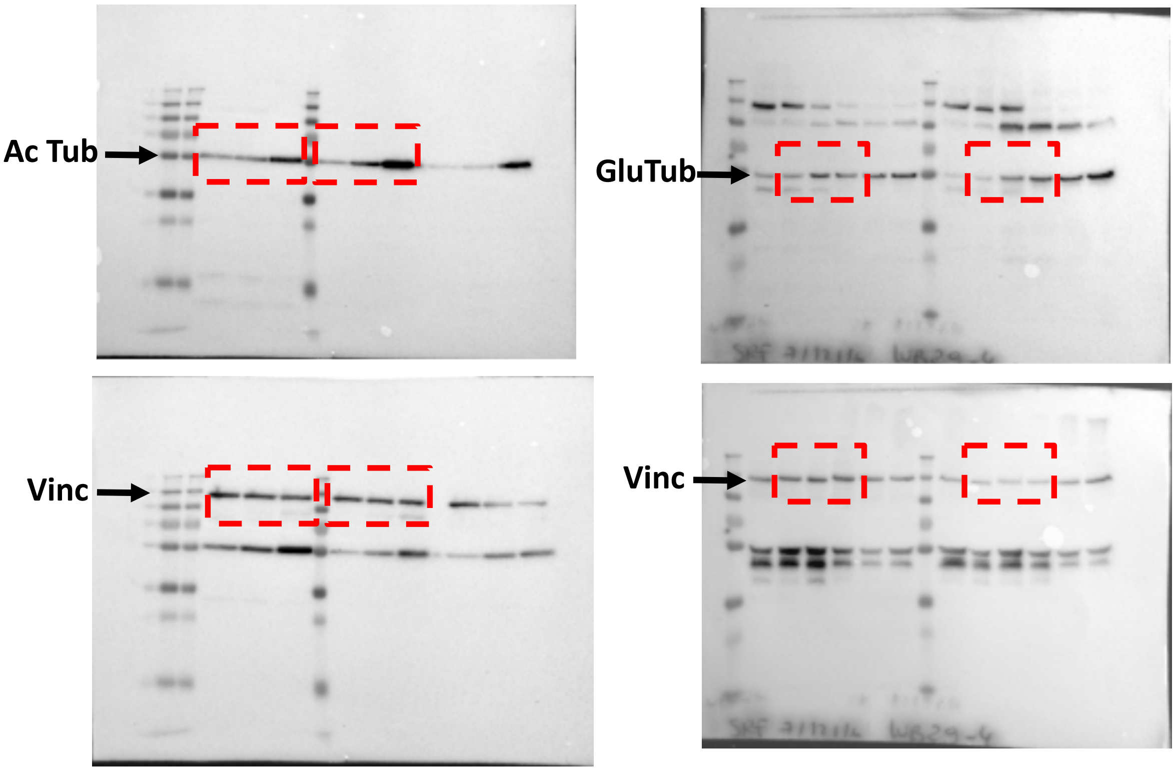

B

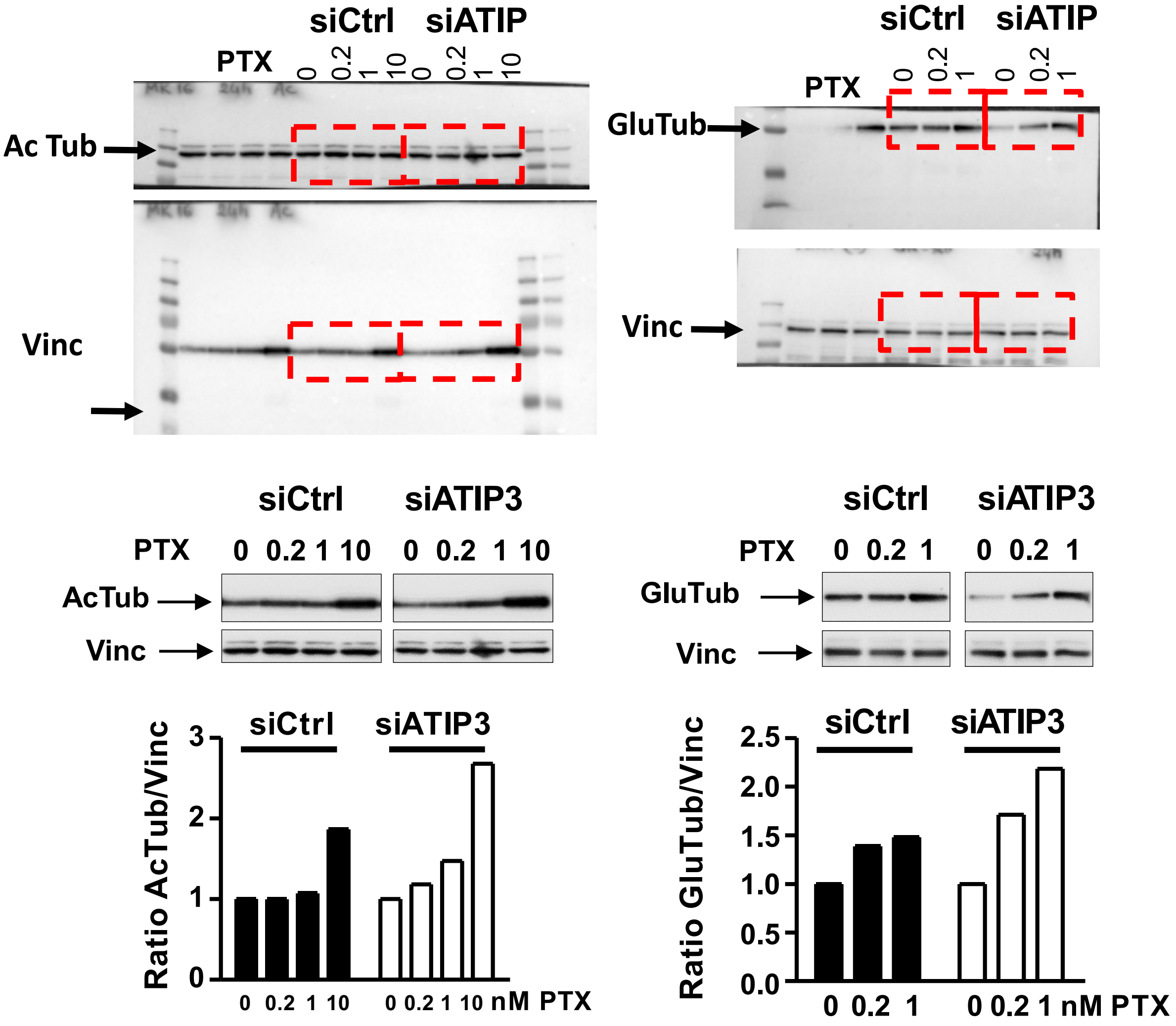

Figure S2. ATIP3 silencing increases the microtubule-stabilizing effects of PTX

A- Uncropped images from Western Blot analysis of acetylated tubulin (AcTub, left panel) and detyrosinated tubulin (GluTub, right panel) in SUM52-PE multicellular spheroids expressing (shCrtl) or not (shATIP3) ATIP3, following 3-day treatment with 10 and 50 nM Paclitaxel (PTX).

B- Western Blot analysis of acetylated tubulin (AcTub, left panel) and detyrosinated tubulin (GluTub, right panel) in 2-dimensional cultures of MDA-MB-231 breast cancer cells expressing (siCrtl) or not (siATIP3) ATIP3, following 24 h treatment with 0.2, 1 and 10 nM Paclitaxel (PTX) as indicated. Vinculin (Vinc) is used as internal loading control. Upper panel: uncropped images. Middle panel: cropped images. Lower panel: quantification. Data were normalized relative to the value of untreated cells (lane 0).

Rodrigues-Ferreira et al., supplemental Fig S3

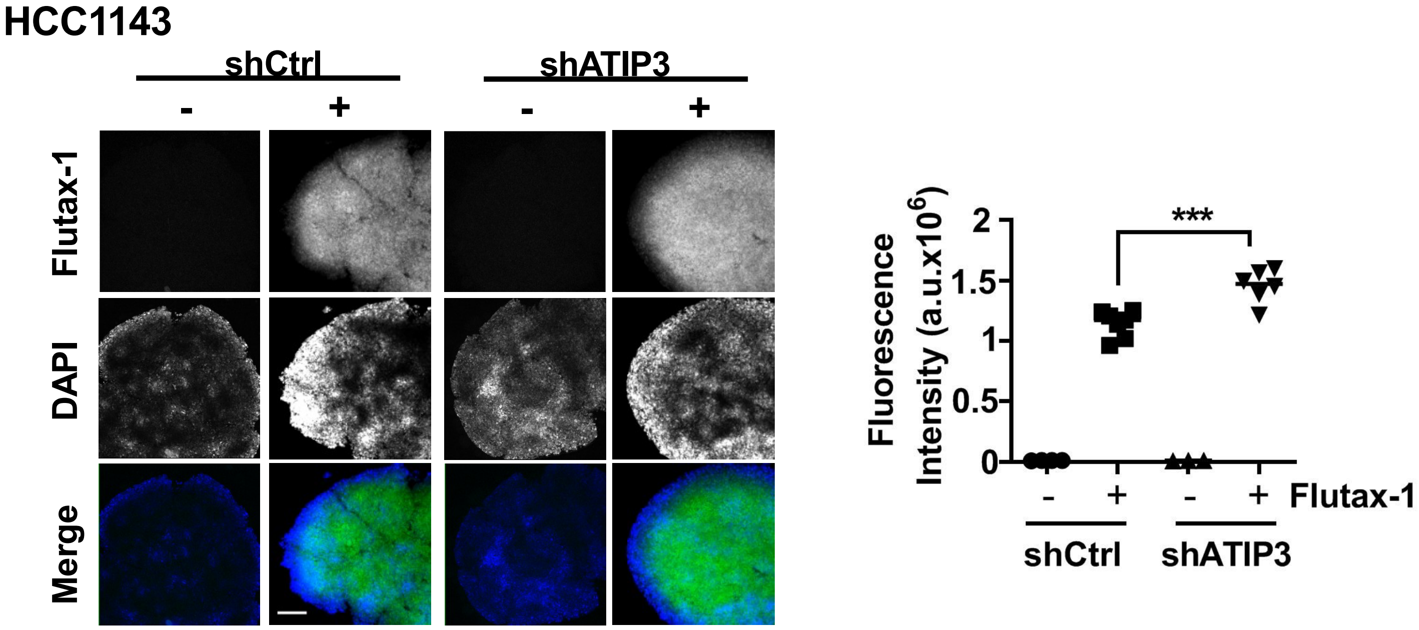

Figure S3. ATIP3 silencing improves the intracellular accumulation of fluorescent Taxol derivative Flutax-1

Representative photographs of fluorescent Taxol derivative Flutax-1 (green) staining in 3-dimensional cultures (MCS) of HCC1143 cells expressing (shCtrl) or not (shATIP3) ATIP3. DNA was stained in blue (DAPI). Fluorescence intensity was measured in each individual MCS and results are plotted in scattered dot plot on the right. a.u : arbitrary units. Obj x20. Scale bar 100μM. *** p=0.0003.

Supplemental Table S1. MTUS1 level and lymph node status in breast cancer patients

	MTUS1 probeset intensities			Lymph Nodes N	
Sample ID	**212093_s_at**	**212095_s_at**	**212096_s_at**	**Pre-treatment**	**Post-treatment**
M106	330,44	379,134	498,342	N0	N0
M107	212,648	356,432	483,247	N+	N+
M108	222,219	225,898	199,482	N0	N0
M111	229,169	203,224	114,96	N+	N0
M113	315,077	410,026	542,41	N0	N0
M116	222,674	179,541	219,512	N+	N0
M117	257,877	285,721	242,212	N+	N+
M120	166,893	264,403	308,182	N0	N0
M121	245,296	257,47	173,665	N0	N0
M123	265,604	342,871	369,086	N+	N0
M126	202,587	174,842	91,41	N0	N+
M128	291,264	347,684	419,267	N0	N0
M129	173,618	257,219	180,169	N0	N0
M130	255,584	282,754	259,079	N+	N+
M133	196,783	277,583	169,991	N0	N0
M135	225,414	258,246	218,257	N0	N+
M136	221,744	229,812	208,759	N0	N0
M139	274,101	288,768	271,182	N0	N0
M141	101,422	154,733	90,3969	N+	N0
M145	139,301	221,19	208,291	N+	N+
M146	186,54	310,882	294,94	N0	N0
M153	258,059	317,605	311,109	N+	N0
M154	218,852	230,511	133,956	N+	N+
M155	203,702	176,109	144,938	N0	N0
M156	256,03	278,478	301,668	N+	N+
M157	281,409	296,432	457,52	N0	N0
M158	249,562	267,101	251,044	N+	N+
M159	191,495	233,683	168,275	N0	N0
M161	252,518	246,922	377,344	N+	N0
M165	199,743	187,567	183,641	N+	N+
M176	224,972	247,019	212,015	N+	N+
M177	198,986	219,31	177,967	N0	N0
M179	213,714	244,982	261,225	N+	N0
M180	246,018	236,605	239,359	N+	N0
M181	246,122	323,765	274,547	N+	N+
M182	273,417	420,782	344,385	N0	N0
M186	230,653	262,69	198,389	N0	N0
M188	287,472	339,798	605,971	N+	N+
M189	170,611	257,821	195,445	N0	N0
M196	307,189	268,708	405,716	N+	N+
M199	202,901	281,156	215,35	N+	N0
M201	321,484	446,264	603,234	N+	N+
M205	187,332	181,369	145,284	N+	N0
M206	222,294	200,998	203,308	N0	N0
M211	193,766	220,483	120,008	N+	N0
M212	250,788	248,73	228,441	N+	N0
M214	213,094	185,339	180,782	N+	N+
M215	239,827	269,171	154,303	N+	N0
M216	226,005	191,768	206,897	N+	N+
M217	291,431	339,997	402,008	N0	N0
M220	158,349	336,234	257,163	N+	N0
M226	222,862	278,555	148,978	N+	N+
M227	233,512	270,489	240,822	N+	N+
M228	177,25	264,727	154,856	N+	N+
M230	145,087	386,185	157,787	N+	N+
M231	221,384	406,594	275,602	N0	N+
M233	258,269	243,814	264,047	N+	N+
M234	267,986	241,003	305,514	N+	N0
M235	194,774	277,916	245,823	N+	N0
M236	214,97	316,365	317,643	N+	N0
M237	177,709	268,455	221,987	N+	N+
M238	239,646	504,459	327,458	N0	N0
M239	235,538	622,59	265,046	N0	N0
M245	372,921	731,232	1009,19	N+	N+

M246	303,893	497,885	570,096	N+	N+
M247	167,825	416,453	171,762	N0	N+
M251	266,382	341,131	385,878	N+	N0
M255	257,135	338,05	281,437	N+	N+
M256	190,775	260,151	327,792	N+	N+
M257	288,665	592,669	426,446	N0	N0
M258	211,287	314,591	420,891	N+	N+
M259	219,024	212,206	174,491	N0	N0
M260	315,671	259,211	285,753	N+	N+
M261	225,171	218,567	230,426	N0	N+
M264	366,745	520,778	751,456	N+	N+
M266	218,892	336,174	192,839	N+	N+
M270	189,768	248,134	241,246	N0	N0
M280	213,858	365,344	234,5	N+	N+
M282	269,596	238,913	226,327	N+	N+
M286	420,721	505,178	681,774	N+	N+
M287	225,569	227,454	255,708	N+	N+
M295	191,843	210,211	141,344	N+	N0
M297	280,124	260,429	303,8	N+	nd
M301	245,224	244,304	291,508	N+	nd
M302	244,443	224,765	217,506	N+	N0
M304	279,783	254,654	302,311	N0	N+
M309	193,937	217,975	172,479	N+	N0
M310	299,45	234,183	213,123	N+	N+
M315	124,396	247,621	134,653	N+	N+
M316	197,389	553,677	260,574	N+	N0
M322	246,202	489,895	457,9	N0	nd
M323	176,126	409,835	419,325	N+	N+
M330	280,246	517,05	422,657	N+	N+
M331	191,527	506,375	126,795	N+	N0
M333	216,046	389,938	174,021	N+	N+
M334	308,674	405,1	481,585	N+	N+
M341	230,197	558,401	773,712	N+	N+
M343	220,799	324,792	399,307	N+	N+
M353	154,627	339,245	385,804	N+	N0
M356	173,664	288,934	384,168	N+	N+
M357	154,343	333,595	306,788	N0	N+
M367	132,84	198,127	149,143	N+	N0
M371	204,554	526,682	191,996	N+	N0
M373	168,785	328,475	482,744	N+	N0
M375	232,039	592,843	630,579	N+	N+
M384	162,98	372,754	314,708	N0	N0
M386	247,203	431,974	432,35	N0	N0
M387	215,955	449,949	298,044	N0	N+
M399	128,592	218,274	151,974	N+	N+
M402	192,764	406,43	163,112	N+	N0
M421	226,562	331,098	441,354	N+	N+
M434	180,619	299,368	306,88	N+	N+
M442	168,016	271,732	274,928	N+	N0
M447	245,696	478,314	568,033	N0	N0
M463	164,165	409,8	143,967	N+	N+
M469	110,345	205,063	154,874	N+	N+
M482	149,92	268,456	328,004	N+	N0
M484	222,626	391,844	493,724	N0	N+
M485	89,5595	134,319	84,7554	N+	N0
M497	109,795	193,043	118,506	N+	N0
M503	134,421	235,687	243,563	N0	N0
M506	261,076	457,685	555,622	N+	N+
M523	234,446	374,442	511,491	N+	N0
M524	99,1607	141,682	92,1711	N+	N0
M525	159,962	376,681	385,291	N0	N0
M534	226,953	371,608	496,319	N+	N+
ML20	211,994	650,696	136,695	N+	N0
PERU01	114,897	373,637	358,269	N+	N+
PERU07	142,185	324,672	181,054	N+	N+
PERU09	166,577	324,007	402,432	N+	N+
PERU11	164,535	318,289	136,07	N+	N+
PERU14	113,067	213,926	126,245	N+	nd
PERU16	150,034	243,697	218,529	N+	nd
